# Relative Sit-to-Stand Power Versus Conventional Sit-to-Stand Time in the Clinical Assessment of Older Adults at Risk of Falls

**DOI:** 10.3390/healthcare14172754

**Published:** 2026-08-31

**Authors:** Elisabet Huertas-Hoyas, Agustín Curiel-Regueros, Rafael García-Molina, Sara García-De-Villa, Eva Rincón-Herrera, Cristina Alonso-Bouzón, Luisa Ruiz-Ruiz, Antonio R. Jiménez-Ruiz, Marta Neira-Álvarez

**Affiliations:** 1 Research Group of Participation, Roles, Occupations and Activities for Community Transformation (PROACT), Physical Therapy, Occupational Therapy, Rehabilitation, and Physical Medicine Department, Rey Juan Carlos University, 28922 Madrid, Spain; 2 Department of Geriatrics, Foundation for Research and Biomedical Innovation of the Infanta Leonor University Hospital (FIIB HUIL), 28031 Madrid, Spain; 3 Department of Geriatrics, Perpetuo Socorro University Hospital, 02006 Albacete, Spain; 4 Center for Biomedical Research Network on Fragility and Healthy Aging (CIBERfes), Instituto de Salud Carlos III, 28029 Madrid, Spain; 5 School of Experimental Sciences and Technology, Rey Juan Carlos University, 28933 Madrid, Spain; 6 Department of Geriatrics, Foundation for Research and Biomedical Innovation of the Getafe University Hospital, 28905 Madrid, Spain; 7 Centre for Automation and Robotics, CSIC-UPM, Arganda del Rey, 28500 Madrid, Spain

**Keywords:** frailty, older adults, sit to stand power, relative muscle power, falls, functional performance, mobility assessment, neuromuscular function

## Abstract

**Highlights:**

**What are the main findings?**

- Higher relative STS power was associated with lower Fried scores and better gait and Timed Up and Go performance.
- A model including relative STS power explained more variance in Fried score than the corresponding model including STS time (39.6% vs. 33.1%).

**What are the implications of the main findings?**

- Relative STS power may complement conventional functional measures by providing additional information on lower-limb neuromuscular performance.
- Its longitudinal and discriminative clinical utility should be confirmed before it is used for individual risk classification.

**Abstract:**

**Background/Objectives**: Conventional time-based sit-to-stand measures may not fully reflect lower-limb neuromuscular capacity. This study examined whether relative sit-to-stand (STS) power provides explanatory capacity compared with STS time in the functional assessment of older adults at risk of falls. **Methods**: A secondary cross-sectional analysis was conducted using data from the publicly available GAIT2CARE database. Participants had a documented history of falls, fear of falling, or stability problems. Absolute, relative, and allometrically scaled STS power were estimated from the five-repetition sit-to-stand test using validated equations. Associations between relative STS power, the Fried phenotype score, and conventional functional measures were examined using Spearman correlations, multiple linear regression, and ordinal logistic regression. **Results**: A total of 127 participants were included, of whom 111 completed the sit-to-stand test and had estimable STS power. Relative STS power was associated with lower Fried scores (ρ = −0.585), faster gait speed (ρ = 0.536), and better Timed Up and Go performance (ρ = −0.533; all *p* < 0.001). After adjustment for age and Timed Up and Go performance, relative STS power remained associated with lower Fried categories (B = −0.635; β = −0.450; *p* < 0.001). The model including relative STS power explained 39.6% of the variance in Fried score, compared with 33.1% for the corresponding model including STS time. Ordinal logistic regression confirmed the association between higher relative STS power and lower Fried categories (estimate = −1.247; *p* < 0.001). **Conclusions**: Relative STS power provides complementary information on lower-limb neuromuscular performance and shows greater explanatory capacity for Fried score than STS time when considered alongside age and Timed Up and Go performance. It may therefore complement, rather than replace, established functional measures in older adults at risk of falls.

## 1. Introduction

Frailty is a geriatric syndrome characterized by a progressive decline in physiological reserves and reduced ability to respond to stressors, which increases the risk of adverse health outcomes in older adults [1,2]. This condition is closely associated with a higher likelihood of falls, functional disability, loss of autonomy, and mortality [3,4]. In particular, older adults at risk of falls constitute a high-risk subgroup, as they often present more advanced physical impairment and greater clinical vulnerability compared with non-fallers [5,6]. Accurate functional assessment is therefore essential to enable early identification of physical frailty and to guide effective preventive and rehabilitative interventions. Among the most commonly used functional measures in clinical practice are the Short Physical Performance Battery (SPPB), gait speed, and sit-to-stand (STS) tests [7,8]. These tools are widely applied due to their simplicity and feasibility; however, they may lack sensitivity to detect early neuromuscular impairments, especially in individuals with mild or preclinical physical frailty [9]. Consequently, reliance on traditional time-based or score-based measures alone may limit the ability to identify subtle functional decline in high-risk older populations.

STS time is one of the most widely used functional measures to assess physical performance; however, this test is not used for the diagnosis of physical frailty. The five-times sit-to-stand (STS) test is included in the Short Physical Performance Battery (SPPB) as a measure of lower-extremity function. Owing to its ease of administration and minimal equipment requirements, it is highly feasible for use in both clinical practice and research settings [8,10]. Nevertheless, this measure has important limitations. By focusing exclusively on task completion time, STS time does not distinguish between force production and movement velocity, nor does it account for differences in body size or movement strategy [11,12]. Furthermore, STS performance may be influenced by compensatory strategies, balance confidence, joint disorders such as knee pain, or psychological factors, potentially masking early neuromuscular deterioration [11,12,13,14]. These limitations may explain the low sensitivity of STS time for detecting the early stages of physical frailty [9,13].

In contrast, muscle power has emerged as a more relevant functional indicator, as it declines earlier and more rapidly than maximal muscle strength with aging [15,16]. Muscle power reflects the capacity to generate force quickly and integrates both force and contraction velocity, which are essential for performing daily functional tasks. Aging-related impairments in neuromuscular activation and contraction speed result in disproportionate reductions in movement velocity and power, even when strength losses are modest [15,16]. In this context, relative sit-to-stand power (relative STS power) has been proposed as a potentially more physiologically meaningful indicator of functional performance compared to time-based measures [17,18].

Previous studies have consistently demonstrated that relative STS power is a valid, reliable, and clinically meaningful indicator of lower-limb neuromuscular function in older adults [15,18,19,20]. Relative STS power has been shown to be strongly associated with mobility limitations, disability, frailty, poor quality of life, and mortality, while providing functionally relevant cut-off points and normative values for clinical interpretation [15,19]. Furthermore, STS-derived power has demonstrated good validity against laboratory-based assessments of lower-limb muscle power and can be accurately estimated using a simple equation based on the five-times sit-to-stand test, making it feasible for routine clinical practice and large epidemiological studies [18,20]. Importantly, unlike time-based outcomes, relative STS power integrates body mass and movement velocity into a physiologically meaningful measure of muscle performance, thereby providing a more direct estimate of the neuromuscular capacity required to rise independently from a chair [14,16,20]. However, few studies have directly compared the predictive performance of relative STS power and STS time, particularly in older adults at risk of falls, a population in whom sensitive functional markers are especially needed [5,6]. Older adults at risk of falls represent a particularly vulnerable population, characterized by greater functional impairment and an increased risk of disability, institutionalization, and mortality, underscoring the need for sensitive and clinically meaningful assessment tools in this group.

Therefore, we hypothesized that relative STS power would show greater explanatory capacity for frailty-related functional performance than conventional STS time in older adults at risk of falls. The overall objective of this study was to compare the explanatory capacity of relative STS power and conventional STS time in relation to frailty-related functional performance. Specifically, we aimed to describe relative STS power values, examine their associations with conventional physical performance measures, compare the association of relative STS power and STS time with the Fried frailty score, and determine whether the association between relative STS power and frailty remained significant after adjustment for relevant covariates.

## 2. Materials and Methods

### 2.1. Study Design and Data Source

This study is a secondary cross-sectional analysis of an existing dataset comprising older adults at risk of falls. All available records corresponding to older adults with a documented history of falls, fear of falling syndrome, or stability problems were included. Data were obtained from the public GAIT2CARE database deposited in Zenodo (DOI: 10.5281/zenodo.15276115). No additional exclusion criteria were applied beyond data availability for the variables of interest.

### 2.2. Participants and Variables

The following variables were extracted from the dataset: age; sex; anthropometric measures, including body mass, height, and body mass index (BMI); Fried phenotype score [1]; overall physical performance assessed using the Short Physical Performance Battery (SPPB) [7]; 4 m walk time and 4 m gait speed [8]; handgrip strength; five-repetition sit-to-stand (STS) time [7,19]; and Timed Up and Go (TUG) time [21].

### 2.3. Sit-to-Stand Performance and Muscle Power Calculation

Sit-to-stand performance was assessed using a standardized chair-stand test with a chair height of 44–45 cm, corresponding to the chair-stand component of the validated Short Physical Performance Battery (SPPB) [7]. STS time was defined as the time, expressed in seconds, required to complete the chair-stand task. Based on this value, STS-derived muscle power parameters were estimated using the validated equations proposed by Alcázar and colleagues [19]. Absolute STS power represents the estimated mean mechanical power generated during the concentric, upward phase of the sit-to-stand movement and is expressed in watts (W). Relative STS power was calculated as absolute power normalized to body mass and is expressed in watts per kilogram (W/kg). Allometric STS power was calculated by normalizing absolute power using an allometric scaling approach to account for differences in body size.

Because the STS time available in the GAIT2CARE dataset was obtained using the SPPB chair-stand protocol, in which timing ends when the participant reaches the standing position on the fifth repetition, the timing procedure differed from the protocol originally used to derive the STS power equations, which includes the return to the seated position after the fifth repetition. Consequently, the STS power estimates in the present study should be interpreted in the context of this methodological difference.

### 2.4. Statistical Analysis

Statistical analyses were performed using IBM SPSS Statistics (version 31.0, IBM Corp., Armonk, NY, USA). Descriptive analyses were conducted for sociodemographic, anthropometric, and functional variables. Quantitative variables were described using means and standard deviations or medians and interquartile ranges, as appropriate. Categorical variables were expressed as absolute frequencies and percentages.

Spearman correlation coefficients were calculated to examine bivariate associations between Fried score and functional, anthropometric, and physical performance variables. Correlations among functional variables were also examined to identify highly interrelated predictors and minimize potential multicollinearity in subsequent multivariable analyses.

Given the ordinal and bounded nature of the Fried score, ordinal logistic regression was specified as the principal multivariable inferential analysis. The proportional odds assumption was assessed using the test of parallel lines. An ordinal logistic regression model was fitted with Fried score as the dependent variable and relative STS power, TUG, and age as predictors.

Complementary multiple linear regression models were performed, treating the Fried score as a continuous outcome, primarily to compare the explanatory capacity of relative STS power and conventional 5STS time using R^2^ and adjusted R^2^ under comparable model specifications. This approach assumes approximately equal spacing between Fried score categories and was therefore interpreted cautiously. Three models were constructed. Model 1 included Fried score as the dependent variable and 5STS time, TUG, age, weight, height, and relative STS power as predictors. Model 2 included relative STS power, TUG, and age as predictors. Model 3 included 5STS time, TUG, and age as predictors. These models were designed to compare the explanatory capacity of relative STS power and conventional 5STS time while minimizing potential multicollinearity.

Predictor selection was based on clinical relevance, bivariate associations, and potential multicollinearity. Grip strength and gait speed were not considered as candidate predictors because they are components of the Fried frailty phenotype and their inclusion would introduce conceptual overlap with the dependent variable. Sex and BMI were not retained in the final comparative models because relative STS power is normalized to body mass, while weight and height were explored in Model 1.

For the linear regression models, R^2^, adjusted R^2^, the F statistic, and its significance level were reported. Individual predictors were interpreted using unstandardized coefficients (B), standardized coefficients (β), t values, and *p* values. Multicollinearity was assessed using tolerance values and variance inflation factors (VIFs), with VIF values within acceptable ranges considered indicative of no relevant multicollinearity. The Durbin–Watson statistic was also examined as an indicator of residual independence.

For the ordinal logistic regression, parameter estimates and *p* values were reported, and model fit was assessed using the likelihood-ratio chi-square test and Nagelkerke R^2^. The proportional odds assumption was evaluated using the test of parallel lines.

The level of statistical significance was set at *p* < 0.05.

### 2.5. AI-Assisted Writing Statement

During the preparation of this manuscript, the authors used ChatGPT (GPT-5.6 Luna, OpenAI, San Francisco, CA, USA) to assist with language editing, formatting, and improvement of scientific clarity. All authors critically reviewed and edited the content and take full responsibility for the integrity and accuracy of the final manuscript.

## 3. Results

### 3.1. Baseline Characteristics

A total of 127 participants were included in the study. Notably, of the initial 127 patients, 16 were unable to perform the sit-to-stand test (STS), resulting in a total of 111 patients for whom power parameters could be estimated. Due to this and other missing data for various variables, the number of observations varied between analyses, reflecting incomplete assessments in specific functional tests. Descriptive characteristics were calculated at the participant level using baseline data to ensure independence of observations. The mean age of the sample was 82.05 ± 5.48 years, and the majority of participants were women (66.7%). Anthropometric characteristics and functional performance measures are presented in Table 1. In total, 64.8% of participants were classified as pre-frail and 31.5% as frail, reflecting a population with substantial frailty-related vulnerability. As a result, all analyses were performed using the data available for each variable.

### 3.2. Correlation Analysis

**Figure 1:**
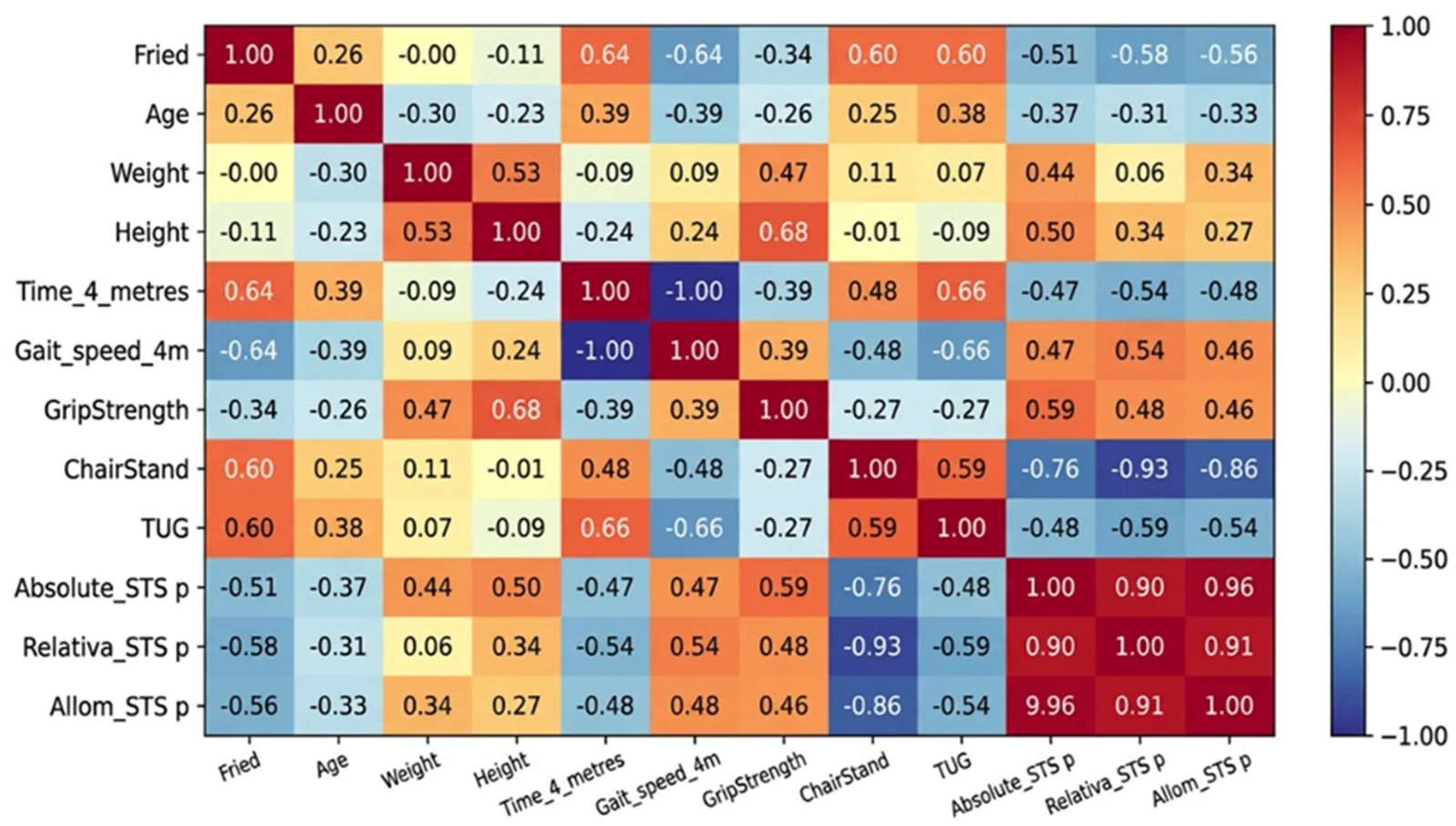
Spearman correlation matrix between STS-derived power metrics and conventional functional measures. Note: Heatmap of Spearman’s rank correlation coefficients (ρ) showing the relationships among Fried score, age, anthropometric variables (weight and height), functional performance measures (5STS time, gait speed, handgrip strength, chair stand test, and Timed Up and Go [TUG]), and STS-derived power metrics (absolute, relative, and allometric). Warmer colors indicate positive correlations, whereas cooler colors indicate negative correlations. Darker color intensity reflects stronger correlation coefficients.

Spearman correlations showed that the Fried score was significantly associated with age and with most of the functional variables analyzed. Fried was positively correlated with age (rho = 0.263; *p* = 0.005), 4 m walk time (rho = 0.642; *p* < 0.001), STS Time (rho = 0.600; *p* < 0.001), and TUG (rho = 0.551; *p* < 0.001). Conversely, Fried was negatively correlated with 4 m gait speed (rho = −0.642; *p* < 0.001), grip strength (rho = −0.341; *p* < 0.001), absolute STS power (rho = −0.505; *p* < 0.001), relative STS power (rho = −0.585; *p* < 0.001), and allometric STS power (rho = −0.560; *p* < 0.001). No significant associations were observed between Fried and either weight or height (Figure 1).

Spearman correlations showed that all STS-derived power metrics were significantly associated with Fried score and conventional functional outcomes (Table 2). Relative STS power showed the strongest correlation with Fried score and STS time performance, while absolute STS power showed the strongest correlation with gait speed. Overall, higher STS-derived power values were associated with lower frailty and better functional performance (Table 2).

### 3.3. Regression Models

Given the ordinal nature of the Fried score, ordinal logistic regression was considered the primary multivariable inferential analysis (Table 3). The model including relative STS power, TUG, and age showed a significantly greater explanatory capacity than the intercept-only model, χ^2^(3) = 54.070, *p* < 0.001, with a Nagelkerke R^2^ of 0.422. The proportional odds assumption was satisfied, χ^2^(9) = 4.341, *p* = 0.888. Relative STS power was negatively associated with higher Fried categories (Estimate = −1.247, *p* < 0.001), whereas TUG was positively associated with higher Fried categories (Estimate = 0.154, *p* = 0.001). Age was not significantly associated with Fried score (*p* = 0.943).

Complementary multiple linear regression models were performed to facilitate comparison of the explanatory performance of relative STS power and conventional STS time. Model 1 included STS time, TUG, age, weight, height, and relative STS power as predictors. Model 2 included relative STS power, TUG, and age, whereas Model 3 included STS time, TUG, and age. This approach allowed comparison of the explanatory contribution of relative STS power with the conventional STS time measure.

In Model 1 of the multiple linear regression analysis (Table 4), Fried score was entered as the dependent variable, while STS time, TUG, age, weight, height, and relative STS power were entered as predictors. The model was statistically significant, F(6103) = 12.085, *p* < 0.001, explaining 41.3% of the variance in Fried score (R^2^ = 0.413; adjusted R^2^ = 0.379). In the adjusted model, relative STS power was negatively and significantly associated with Fried score (B = −0.840; β = −0.595; *p* < 0.001), whereas TUG showed a positive significant association (B = 0.044; β = 0.241; *p* = 0.010). Age, weight, height, and STS time were not significantly associated with Fried score (*p* > 0.05). VIF values ranged from 1.260 to 5.020, suggesting no critical multicollinearity for most predictors, although relative STS power showed a borderline value.

Model 2 of the multiple linear regression analysis (Table 4), which included relative STS power, TUG, and age as predictors of Fried score, was statistically significant, F(3106) = 23.149, *p* < 0.001, explaining 39.6% of the variance in Fried score (R^2^ = 0.396; adjusted R^2^ = 0.379). In the adjusted model, relative STS power was negatively associated with Fried score (B = −0.635; β = −0.450; *p* < 0.001), whereas TUG showed a positive significant association (B = 0.046; β = 0.251; *p* = 0.005). Age was not significantly associated with Fried score (*p* = 0.621). VIF values ranged from 1.133 to 1.408, indicating the absence of relevant multicollinearity.

Model 3 of the multiple linear regression analysis (Table 4), which included STS time, TUG, and age as predictors of Fried score, was statistically significant, F(3106) = 17.453, *p* < 0.001, explaining 33.1% of the variance in Fried score (R^2^ = 0.331; adjusted R^2^ = 0.312). In the adjusted model, TUG (B = 0.058; β = 0.316; *p* < 0.001) and STS time (B = 0.047; β = 0.315; *p* < 0.001) were positively associated with Fried score, indicating that poorer functional performance was related to higher levels of frailty. Age was not significantly associated with Fried score (*p* = 0.194). VIF values ranged from 1.079 to 1.296, indicating the absence of relevant multicollinearity.

When comparing the multiple linear regression models designed to examine functional performance associated with Fried score, the model including relative STS power, TUG, and age showed a greater explanatory capacity than the model including STS time, TUG, and age. Specifically, the model with relative STS power explained 39.6% of the variance in Fried score, with an adjusted R^2^ of 0.379, whereas the model with STS time explained 33.1% of the variance, with an adjusted R^2^ of 0.312. Both models were statistically significant; however, the model including relative STS power showed greater explanatory capacity.

Furthermore, in the model including relative STS power, this variable showed a negative and significant association with Fried score (B = −0.635; β = −0.450; *p* < 0.001), indicating that higher relative power values were associated with lower levels of frailty. In the alternative model including STS time, this variable was also significantly associated with Fried score (B = 0.047; β = 0.315; *p* < 0.001), although its relative predictive weight was lower and the overall model fit was poorer. These findings indicate that, within the present sample, relative STS power showed greater explanatory capacity for Fried score than STS time when both were considered in otherwise comparable models. The strong inverse correlation observed between STS time and relative STS power was expected, as relative STS power is mathematically derived from STS time. For this reason, Model 1 was considered primarily exploratory, allowing the simultaneous assessment of all variables while monitoring multicollinearity through VIF values. To provide a more appropriate comparison of the explanatory value of each functional metric, Models 2 and 3 were intentionally constructed as separate, non-overlapping models. This strategy minimized the impact of collinearity and allowed direct comparison of the variance explained by relative STS power and conventional STS time under otherwise comparable conditions. Because relative STS power is mathematically derived from STS performance and is therefore strongly related to STS time, the comparison between Models 2 and 3 was interpreted as a comparison of explanatory capacity rather than as evidence of incremental clinical validity or added clinical value.

## 4. Discussion

The present study aimed to compare the explanatory capacity of relative STS power and conventional STS time in the evaluation of older adults at risk of falls. Specifically, we sought to describe relative STS power values in this population, examine their associations with conventional physical performance measures, compare the association of relative STS power and STS time with the Fried frailty score, and determine whether the association between relative STS power and frailty remained significant after adjustment for relevant covariates. Overall, our findings showed that higher relative STS power values were associated with lower frailty levels and better functional performance. Moreover, the model including relative STS power showed greater explanatory capacity for Fried score than the model including STS time, indicating greater explanatory capacity for Fried score within the present sample and supporting its potential role as a complementary functional indicator.

In descriptive terms, the mean relative STS power observed in our sample was 2.19 ± 0.76 W/kg. However, this value should be interpreted cautiously because the STS timing protocol used in the source dataset differed from the protocol underlying the equations used to estimate STS power. Therefore, direct comparison of the absolute power values observed in the present sample with previously published normative values or clinically relevant thresholds should be avoided. Rather than interpreting the absolute values as directly transferable clinical reference values, the present findings are primarily relevant to the observed associations between relative STS power and frailty-related functional performance within this sample. Multiple linear regression models were then performed using Fried score as the dependent.

These findings are consistent with the body of evidence generated by Alcázar and colleagues, who have consistently demonstrated that STS-derived relative power is a more representative measure of lower-limb neuromuscular function than the time required to complete the sit-to-stand test. In a large multicenter European study, Alcázar et al. established normative values and clinically relevant cut-off points for relative STS power, demonstrating strong associations with aging, functional limitations, disability, and frailty [17]. Subsequently, the same research group validated simplified equations for estimating muscle power from the five-repetition sit-to-stand test, showing high agreement with laboratory-based reference methods and confirming that this approach provides a valid, portable, and low-cost alternative for estimating lower-limb muscle power in older adults [18,19]. More recently, the accuracy of these equations has been further confirmed in older adults with different functional levels using sensor-based technologies, reinforcing the clinical applicability of relative STS power for both research and routine clinical practice [20].

From a physiological perspective, these findings are consistent with the conceptual differences between both measures. Whereas STS time only reflects the time required to complete the task and may be influenced by multiple factors, including balance, compensatory strategies, joint pain, and motor coordination, relative STS power incorporates the body mass displaced and the velocity of movement to estimate the mechanical power generated during the sit-to-stand task. Since muscle power is considered one of the main determinants of physical performance and functional independence during aging [15], it is reasonable that relative STS power demonstrates a greater ability to identify the neuromuscular impairment associated with frailty than a measure based solely on task completion time. In this regard, recent publications from Alcázar’s group have consolidated the concept of sit-to-stand power as a functional biomarker of neuromuscular capacity, supporting the use of relative power as the most clinically meaningful expression because it accounts for the influence of body mass on functional performance [20,22].

The relationship observed between relative STS power and conventional mobility measures also deserves consideration. In the present study, higher Relative STS power values were associated with faster gait speed and better TUG performance, suggesting that lower-limb relative power is closely related to global mobility capacity. This finding is consistent with previous studies showing that STS-derived relative power is associated with mobility limitations, frailty, and functional performance in older adults [17,20]. However, these measures should not be interpreted as interchangeable. Gait speed is widely considered an integrated marker of health and functional status in older adults, whereas the TUG reflects basic functional mobility involving chair rising, walking, turning, and sitting down [8,23]. In contrast, relative STS power provides a more specific estimate of the neuromuscular capacity required to generate force rapidly during the chair-rise task [14,17]. Therefore, relative STS power may complement, rather than replace, established mobility measures by providing additional information on lower-limb power generation in older adults at risk falls.

Finally, our findings extend previous evidence by directly comparing the explanatory capacity of relative STS power and conventional 5STS time using the Fried phenotype score as the reference outcome. Although STS-derived relative power has previously been associated with mobility limitations, disability, frailty, and mortality [16,19,20,21], few studies have compared both measures within multivariable models. In the present study, the model including relative STS power, TUG, and age explained 39.6% of the variance in Fried score, compared with 33.1% for the model including 5STS time, indicating greater explanatory capacity for relative STS power. This finding may be explained by its integration of body mass and movement velocity, which provides a more physiologically meaningful estimate of lower-limb neuromuscular performance than task completion time alone [14,15,16]. From a clinical perspective, relative STS power may complement established functional measures by characterizing lower-limb neuromuscular performance from a different measurement perspective [5,6]. Nevertheless, relative STS power should not be regarded as a replacement for gait speed, TUG, or other validated functional tests, but rather as a feasible complementary metric that may support a more comprehensive functional assessment. From a practical perspective, the additional information provided by relative STS power may help distinguish between patients with similar STS times but different neuromuscular capacities due to differences in body mass. Importantly, calculating relative STS power does not require additional equipment beyond the conventional five-times sit-to-stand test, as it can be estimated using validated equations or readily available digital tools. Therefore, incorporating this metric into routine geriatric assessment may provide a more refined characterization of lower-limb neuromuscular performance without substantially increasing clinical workload. Future longitudinal studies should determine its prognostic value and evaluate its clinical utility for risk stratification, individualized exercise prescription, and monitoring of neuromuscular recovery in older adults at risk of falls.

Several limitations should be acknowledged. First, the STS timing protocol used in the GAIT2CARE dataset differed from that underlying the equations used to estimate STS power: timing ended when the participant reached the standing position on the fifth repetition in the SPPB protocol, whereas the original power protocol includes the return to the seated position. Because STS time contributes directly to the power estimation, this discrepancy may affect the calculated power values. Therefore, absolute relative STS power values should not be directly compared with published normative values or clinical thresholds derived using the original protocol. In addition, given the strong correlation between relative STS power and STS time (ρ = −0.930), the higher variance explained by the relative STS power model should be interpreted as greater explanatory capacity within the present sample, rather than as evidence of incremental clinical validity or superiority over STS time. Second, although linear regression was used to compare the explanatory performance of relative STS power and STS time, the Fried score is an ordinal and bounded outcome. Treating it as continuous assumes approximately equal spacing between score values. To address this limitation, ordinal logistic regression was used as the principal inferential analysis, and the proportional odds assumption was satisfied. The linear models were therefore considered complementary analyses for comparing explanatory performance. Future prospective studies using standardized STS protocols are needed to establish the prognostic and clinical utility of relative STS power.

As a future line of research, the clinical utility of relative STS power should be evaluated for individualizing exercise programs and monitoring neuromuscular recovery in older adults at risk of falls.

## 5. Conclusions

Relative STS power showed greater explanatory capacity for Fried score than conventional 5STS time when considered alongside age and TUG performance. This finding should be interpreted as greater explanatory capacity within the present sample rather than as evidence of incremental clinical validity or superiority over conventional STS time. The association between relative STS power and lower Fried categories was also confirmed using ordinal logistic regression. Relative STS power may therefore be considered a feasible complementary indicator of lower-limb neuromuscular performance in older adults at risk of falls. However, further longitudinal and prospective studies using standardized testing protocols are required to establish its prognostic and clinical utility.

## Figures and Tables

**Table 1:** Baseline characteristics of participants at baseline (N = 111).

Variable	Mean ± SD
Age (years)	82.06 ± 5.48
Weight (Kg)	67.93 ± 12.54
Height (m)	1.55 ± 0.08
BMI (kg/m^2^)	27.73 ± 4.41
Fried	
Fit	3.6%
Pre-frail	64.8%
Frail	31.5%
Time 4 metres (s)	6.77 ± 3.05
Gait Speed (m/s)	0.71 ± 0.20
Handgrip strength (Kg)	18.43 ± 7.72
TUG time (s)	13.42 ± 5.40
STS_Time (s)	15,57 ± 7.41
Absolute STS power (W)	149.49 ± 62.12
Relative STS power (W/Kg)	2.19 ± 0.76
Allometric STS power (W/m^2^)	60.68 ± 22.29

Note: Values are presented as mean ± standard deviation (SD). Values correspond to initial participant-level data. Only n = 111 patients were able to complete the Sit to Stand test.

**Table 2:** Spearman correlations between STS-derived power metrics and conventional functional outcomes.

	Fried	STS Time	Gait_Speed_4m	TUG
Absolute STS power	−0.505 **	−0.764 **	0.470 **	−0.421 **
Relative STS power	−0.585 **	−0.930 **	0.536 **	−0.533 **
Allometric STS power	−0.560 **	−0.860 **	0.479 **	−0.477 **

Note: Values are Spearman’s rank correlation coefficients (ρ). STS, sit-to-stand. All correlations were statistically significant at ** *p* < 0.001.

**Table 3:** Ordinal logistic regression analysis of functional variables associated with Fried score.

	Estimate	SE	Wald χ^2^	Sig.
Age	0.003	0.036	0.005	0.943
TUG	0.154	0.047	10.695	0.001
Relative STS power	−1.247	0.331	14.163	<0.001

Note: Dependent variable: ordinal Fried score. Link function: logit. Model fit: χ^2^(3) = 54.070, *p* < 0.001; Nagelkerke R^2^ = 0.422. The proportional odds assumption was met, χ^2^(9) = 4.341, *p* = 0.888.

**Table 4:** Multiple linear regression analysis of functional variables associated with Fried score.

*Model 1*	B	β	t	Sig.
Age	0.008	0.043	0.508	0.612
Weight	−0.007	0.008	−0.844	0.401
Height	2.310	0.193	1.713	0.09
TUG	0.044	0.241	2.633	0.01
Relative STS power	−0.840	−0.595	−3.521	<0.001
STS time	−0.014	−0.097	−0.644	0.521
***Model 2***				
Age	0.008	0.040	0.496	0.621
TUG	0.046	0.251	2.863	0.005
Relative STS power	−0.635	−0.450	−5.026	<0.001
***Model 3***				
Age	0.021	0.108	1.307	0.194
TUG	0.058	0.316	3.492	<0.001
STS time	0.047	0.315	3.532	<0.001

Note: Dependent variable: Fried score. Model 1: R^2^ = 0.413; adjusted R^2^ = 0.379; model *p* < 0.001; Model 2: R^2^ = 0.396; adjusted R^2^ = 0.379; model *p* < 0.001; Model 3: R^2^ = 0.331; adjusted R^2^ = 0.312; model *p* < 0.001.

## Data Availability

The data analyzed in this study are publicly available in the Zenodo repository as part of the GAIT2CARE dataset (DOI: https://doi.org/10.5281/zenodo.15276115).

## Abbreviations

The following abbreviations are used in this manuscript:

STS	Sit-to-Stand
TUG	Timed up and go
SPPB	Short Physical Performance Battery
